# Current knowledge, attitude and behaviour of hand and food hygiene in a developed residential community of Singapore: a cross-sectional survey

**DOI:** 10.1186/s12889-015-1910-3

**Published:** 2015-06-21

**Authors:** Junxiong Pang, Shao Wei Jonathan Lumen Chua, Liyang Hsu

**Affiliations:** Centre for Infectious Disease Epidemiology and Research, Saw Swee Hock School of Public Health, National University of Singapore, Singapore, Singapore; Department of Medicine, Yong Loo Lin School of Medicine, National University of Singapore, Singapore, Singapore

**Keywords:** Knowledge, Practices, Attitudes, Hand hygiene, Food hygiene, Noncompliant and complacent behaviour, Diarrhoea

## Abstract

**Background:**

Diarrhoea incidence has been increasing progressively over the past years in developed countries, including Singapore, despite the accessibility and availability to clean water, well-established sanitation infrastructures and regular hygiene promotion. The aim of this study is to determine the current knowledge, attitude and behaviour of hand and food hygiene, and the potential risk factors of diarrhoea in a residential community of Singapore.

**Methods:**

A cross-sectional study was conducted within a residential area in the west of Singapore from June to August 2013. A total of 1,156 household units were randomly sampled and invited to participate in an interviewer-assisted survey using standardised questionnaires. Descriptive, univariate and multivariate analyses were performed using descriptive statistics, Fisher’s Exact test and multivariate logistic regression modelling, respectively. R program was used for all statistical analysis. All tests were conducted at 5 % level of significance with 95 % confidence intervals (CI) reported where applicable.

**Results:**

A total of 240 units (20.8 %) consented and responded to the survey invitation. About 77 % of the expected knowledge and attitude were observed in at least 80 % of the participants, compared to only about 31 % of the expected behaviours and practises. Being single [adjusted odds ratio (AOR) = 2.29; 95 % CI = 1.16-4.48], having flu in the past six month (AOR = 3.24; 95 % CI = 1.74-6.06), preferred self-medication (AOR = 2.07; 95 % CI = 1.06–4.12) were risk factors of diarrhoea. Washing hands with water before attending to children or sick persons (AOR = 0.30; 95 % CI = 0.11–0.82), washing hands with water (AOR = 0.16; 95 % CI = 0.05–0.45) and water with soap (AOR = 0.29; 95 % CI = 0.12–0.72) after attending to children or sick persons, and hand washing between 30 s to a minute (AOR = 0.44; 95 % CI = 0.20-0.90) were protective factors against diarrhoea.

**Conclusions:**

Good knowledge and attitude of the participants did not positively translate into high compliance and motivation to perform good hygiene practices. This observation may have resulted in a significant extent on the increasing diarrhoea incidences. Current interventions may be improved with more active community partnership among the residents, schools and the relevant social organizations, to raise awareness on the importance of compliance to good hygiene practices, and the risk factors of diarrhoea. A large case–control study would be required to validate these findings in future.

**Electronic supplementary material:**

The online version of this article (doi:10.1186/s12889-015-1910-3) contains supplementary material, which is available to authorized users.

## Background

Diarrhoea is one of leading causes of morbidity and mortality across all age groups and regions of the world, particularly in the less developed communities and children below the age of five. Globally, diarrhoea episodes in children under the age of five, are estimated at about 1.7 billion of which 36 million were severe cases [1, 2]. Among which there were an estimated 700 000 deaths annually, which is lower than in 2005 where estimates range between 1.6 million and 2.6 million [1, 3, 4]. Furthermore, diarrhoea poses a substantial burden accounting for approximately 2.8 billion diarrhoea episodes among older children, adolescents, and adults [2], even in the developed communities [5–7]. Infection can be spread through contaminated food, drinking water as well as from person-to-person contact due to poor hygiene [1]. However, the main cause of diarrhoea in a developed community is usually due to either foodborne or person to person transmission [8–12]. Diarrhoea can be significantly reduced through improvements in drinking water, sanitation facilities, hygiene knowledge and practices [11, 13, 14]. In addition, well-structured campaigns to improve hygiene knowledge and practices have been shown to be effective in the prevention of diarrhoea disease transmission in clinical settings [15] as well as in the less developed communities [16, 17].

Even though there were much accessibility and availability of well-established infrastructures such as proper sanitation and clean water facilities with soap, and the regular health campaigns/promotions on good hygiene practises in the developed residential communities, there has been gradual increase of acute diarrhoea illnesses and food poisoning notifications over the past years, resulting in significant public health burden [5–12, 18]. As an example, there were a total of 124,292 acute diarrhoea illnesses reported in the Singapore community in 2011, which is an increase of 10.3 % compared to 2010 [19]. Children under the age of five are most affected, which accounted for 47.4 % and 28.1 % of cases infected by *C. enteritis* and *S. enteritidis*, respectively in 2011 [19]. As such, it is critical to understand the current knowledge, attitude and behaviour of good hygiene and its impact on the increased diarrhoea illness in a residential community of a developed country. In addition, most of the current understanding of the knowledge, attitude and behaviour on hygiene was significantly focused on less developed communities [20–24], and very limited in the well-developed communities [11, 25]. Therefore, we aim to determine the current knowledge, attitude and behaviour of good hygiene as well as risk factors of diarrhoea disease in a developed community in Singapore, where clean water and soap are easily available and affordable. The study findings will also help to better inform and guide surveillance and prevention policies and strategies to reduce the public burden of diarrhoea diseases.

## Methods

### Study area and design

An interviewer-assisted cross-sectional survey was conducted in a developed typical residential heartland located in the west of Singapore, from early-June 2013 to early-August 2013. The short duration of this cross-sectional study is to minimise any bias that might have been introduced over a longer duration of the study. For example, the longer the interviewers were present around the neighbourhood, the more likely it may arouse interest among the residents on this study that may indirectly influence their choices during the surveys. In addition, the short duration is an optimal strategy to avoid any form of public campaign or events that may be held which the study team may not be aware of and may indirectly influence the results of this study. This area is one of the furthest residential areas situated away from the central business district (about 18 km) that can truly represent a residential heartland environment in Singapore [11, 18]. In total, the three largest zones (Zone A, B, C), which cover about 6000 residential units, were been selected for the survey. Four Housing Development Board (HDB) blocks from each zone were selected by simple random sampling technique using lottery method from the list of HDB blocks found in each zone. All the units in these blocks were invited to participate in the survey from door-to-door. The questionnaire was designed to tackle the behaviour of the participants first before we questioned them on their knowledge and attitude towards handwashing. This is to ensure that the participants’ responses on their hygienic behaviour would not be biased by the questions on knowledge and attitude.

### Participants and data collection

Participants were selected based on the hierarchy order of the household depending on who is present at the time of the interview. The selection order was from master or mistress of the household, to grandparents or relatives who stay with the family, and children who are above 18 years of age. This strategy also aims to have respondents who had the greatest awareness on the day-to-day household hygiene practices at the time of survey to answer some of the relevant questions in the survey. The surveys were collected over the weekends during daylight hours to increase the chance of engaging the participants invited. Each interview lasted about 25 min on average. Participants were reported having diarrhoea and flu if the participant had at least one episode in the past six months. Diarrhoea is defined by the World Health Organisation of passage of loose or watery stools at least three times in a 24 h period. Flu is defined as having fever, runny nose and sore throat. National data from the HDB [26] and Ministry of Health, Singapore [27] were used as a guiding reference to show if the current subpopulation is a good representative of the general population of Singapore (Table 1).

**Table 1:** Demographics of study population

Demographics	Overall (%) [N = 240]	National HDB data [26] (%)
**Median Age**	41.5	39
Interquartile Range	32-52	
**Gender**		
Female	122 (50.8)	(51.2)
**Marital Status**		
Married	184 (76.7)	(76.5)
**Ethnicity**		
Chinese	159 (66.3)	(73.5)
Malay	30 (12.5)	(15.6)
Indian	32 (13.3)	(8.9)
Others	19 (7.9)	(2)
**Citizenship**		
Singaporean	182 (75.8)	(82)
**Education** ^**Ɨ**^		
Post-Secondary	120 (50)	(51.4)
**Occupation**		
Employed	158 (65.8)	(64)
**Household members**		
<3	37 (15.4)	(28.8)
3-5	145 (60.4)	(63.8)
>5	58 (24.2)	(18.4)
**At least 1 Child <5 Years Old**	55 (22.9)	(5.1)
**Residence**		
2-3 Room	29 (12.1)	(23.7)
4 Room	133 (55.4)	(41.1)
5 Room-Executive	78 (32.5)	(35.2)
**Diabetes**	11 (4.6)	(11.3)^+^
**Hypertension**	34 (14.2)	(23.5)^+^
**Asthma**	4 (1.67)	-
**Allergy**	9 (3.8)	-
**Smoker**	48 (20)	(14.3)^+^
Diarrhoea* ^**#**^	60 (25)	-
Flu*^	73 (30.4)	-

^Ɨ^Post-Secondary refers to individuals with qualification higher than GCE ‘O’ level certification (i.e. GCE ‘A’ level, diploma, degree)

*Participants who self-reported having at least one episode of diarrhoea or flu, respectively, in the past six months

#Diarrhoea as defined by the World Health Organisation of passage of loose or watery stools at least three times in a 24 h period

^Flu is defined as having fever, runny nose and sore throat

+Based on year 2010, Ministry of Health, Singapore [27]

### Data management and analysis

To ensure quality of data, standardized checklist and structured questionnaires was used. Pre-test was done on 25 participants out of the study area and necessary correction was done accordingly. Intensive training was given to six interviewers and supervisor for one day on how to approach study participants, and on how to use the questionnaire. Supervision was done by a supervisor on a regular routine. The collected data was checked for completeness, accuracy and clarity by the supervisor. Appropriate measure was taken on time for completeness before data entry. Data clean up and cross-checking was done before analysis by an independent member who was not involved in data entry. Each completed questionnaire was checked visually for completeness before coded for data entry. A scoring system was utilised to combine sub-questions together for group analysis. Question 4, 5, 19, 20, 21 had multiple parts and a score was given to each answer. In question 4, the response ‘soap’ would be given a score of 2, the response ‘water’ would be given a score of 1 and the response ‘none’ would be given a score of 0. For question 19 and 20, the option with a rank 1 would be given a score of 2, a rank 2 would be given a score of 1 and a score of 0 was given to the option ranked 3. The score for each subquestion was then added up to derive the total score value for each individual and was used in further analysis. The score for Question 21 was determined by the number of ticks on the options of positive action. R version 3.0.1 (R Core Team (2013) Vienna, Austria URL: http://www.R-project.org/) was used for all statistical analysis. Fisher’s Exact Test was used to test for significance with all categorical variables, to identify possible confounders. Generalised linear models were used for multivariate logistical regression to elucidate association and calculation of crude and adjusted odds ratio values. Age, marital status and having flu over the past 6 months were statistically significant on fisher’s exact test and were used to adjust the crude odds ratio in the multivariate logistical regression model. All tests were conducted at the 5 % level of significance with P-value and corresponding 95 % confidence intervals reported where applicable.

Ethical clearance was obtained from the National University of Singapore Institutional Review Board. The purpose of study was well explained to the study participants and informed consent was obtained. Confidentiality was maintained at all levels of the study by avoiding use of name and other identifiers. Participants’ involvement in the study was on voluntary basis; participants who were unwilling to participate in the study and those who wish to quit their participation were informed to do so without any restriction.

## Results

In total, 1,156 units were visited and invited to participate in the survey. There were 707 (61.2 %) units either not at home or not responsive, even on second attempt. Out of the 1,156 units, 240 units (20.8 %) consented and completed the survey, and 18 % rejected participation in the survey. The median age of the participants was 41.5 years old [Interquartile range (IQR): 32–52], 50.8 % was female, 76.7 % was married, 66.3 % was Chinese, 75.8 % was Singaporean, and 55.4 % stayed in a four room flat (Table 1). In addition, 50 % of the participants had post-secondary education (defined as higher than GCE ‘O’ level), 40 % had at least one comorbidity, and the most common comorbidity was hypertension (14.2 %; Table 1). The majority of the participants (60.4 %) had three to five residing members, and 22.9 % had at least a child below the age of five. Furthermore, 25 % of the participants self-reported having at least one diarrhoea episode in the past six month period before the survey day, and about 30 % self-reported having at least one flu episode over the same period (Table 1). The sampled population had similar demographic profiles as the national population (Table 1).

### Hand hygiene knowledge, attitude and behaviour

About 92.5 % reported washing their hands four or more times in a day, while 71.2 % reported washing their hands with soap four or more times in a day (Table 2). Only 6.3 % reported washing their hands with alcohol disinfectant four or more times in a day. The median score on whether they washed their hands on the specified occasions was 21 out of the maximum score of 28 (IQR = 17–23; Table 2). Only 48.8 % washed their hands with soap before meals, and only 58.8 % washed their hands before handling food or cooking. In addition, only 55.8 % and 61.3 % washed their hands with soap before and after attending to a child or sick person, respectively (Table 2). Furthermore, there were only 86.3 % reported washing their hands with soap after defecation, and only 72.5 % washed their hands with soap after a toilet trip.

**Table 2:** Questionnaires and the response

Questions*		(N = 240)	%
**Hand Hygiene - Practice and Behaviour**			
Q1 How often do you wash your hands a day?			
Never		0	0.0
1-3 times		18	7.5
4-8 times		106	44.2
>8 times		116	48.3
Q2 How often do you wash your hands with soap a day?			
Never		4	1.7
1-3 times		65	27.1
4-8 times		110	45.8
>8 times		61	25.4
Q3 How often do you wash your hands with alcohol-based disinfectant a day?			
Never		**192**	**80.0**
1-3 times		33	13.8
4-8 times		9	3.8
>8 times		6	2.5
Q4^Ɨ^ Do you wash your hands….	Median Score (IQR)	21	17-23
A. When your hands are visibly dirty?	Response: Soap	**211**	**87.9**
B. Before eating?	Response: Soap	117	48.8
C. Before handling food or cooking?	Response: Soap	141	58.8
D. After handling food or cooking?	Response: Soap	179	74.6
E. After defecation?	Response: Soap	**207**	**86.3**
F. After a toilet trip?	Response: Soap	174	72.5
G. After an outdoor physical activity?	Response: Soap	176	73.3
H. Before attending to a child or sick person?	Response: Soap	134	55.8
I. After attending to a child or sick person?	Response: Soap	147	61.3
J. After sneezing or coughing?	Response: Soap	103	42.9
K. After handling pets?	Response: Soap	172	71.7
L. With preference of using	Response: Liquid Soap	**196**	**81.7**
M. For about…	Response: >30s	111	46.3
Q5 Do you follow…			
Step 1 of the 8 steps of hand washing?		**211**	**87.9**
Step 2 of the 8 steps of hand washing?		176	73.3
Step 3 of the 8 steps of hand washing?		152	63.3
Step 4 of the 8 steps of hand washing?		82	34.2
Step 5 of the 8 steps of hand washing?		74	30.8
Step 6 of the 8 steps of hand washing?		64	26.7
Step 7 of the 8 steps of hand washing?		103	42.9
Step 8 of the 8 steps of hand washing?		180	75.0
Q6 Do you feel it is troublesome washing your hands with soap?		27	11.3
Q7 Is hand washing with soap a habit for you?		**207**	**86.3**
Q8 Do you think you will encourage your family members and friends to pick up hand washing with soap, if they have not?		187	77.9
Q9 Do you think you will wash your hands with soap more often during disease outbreaks like the previous H1N1 or SARS?		189	78.8
Q10 Do you cover your mouth when you sneeze?		**223**	**92.9**
Q11 Do you wear a mask in public when you are sick?		75	31.3
**Food Hygiene - Practice and Behaviour**			
Q12 Do you keep fresh food such as vegetables and meat separately in the refrigerator with the appropriate temperature?		**232**	**96.7**
Q13 Do you wash your food properly before cooking?		**240**	**100.0**
Q14 Do you have a chopping board that is only for vegetables, and another one just for meat?		136	56.7
Q15 Do you check the expiry date on the food packaging before purchasing or cooking the food?		**226**	**94.2**
Q16 Do you ensure the food is well-cooked before consumption?		**236**	**98.3**
Q17 Do you leave cooked food at room temperature for longer than two hours?		118	49.2
Q18 Do you reheat leftover food before consumption?		**212**	**88.3**
Q19Do you choose hawker stalls based on…			
Their cleanliness grades awarded by NEA		47	19.6
The good recommendation of their food		98	40.8
The cleanliness of the stalls and the hygiene measures taken by their chef(s)		95	39.6
Q20 Do you choose restaurants based on…			
Their cleanliness grades awarded by NEA		89	37.1
The good recommendation of their food		151	62.9
Q21 Will you provide feedback to the….			
Restaurant or hawkers or food handlers if you have diarrhoea after consuming their food		83	34.6
NEA/MOH if you have diarrhoea after consuming food at a hawker or restaurant		47	19.6
None of the above		140	58.3
Q22 Would it be useful to have a web-portal or apps that you can report your diarrheal case?		152	63.3
Q23 When you have diarrhoea do you prefer to….			
Self-medicate		89	37.1
Visit a GP		118	49.2
Visit polyclinic		7	2.9
Visit the hospital		26	10.8
**Hand Hygiene - Knowledge and Attitude**			
Q24 Do you agree that washing your hands with soap can help to reduce the spread of diseases, like HFMD and flu?		**232**	**96.7**
Q25 Do you agree that washing your hands with soap can protect you from diarrheal diseases?		**195**	**81.3**
Q26 Do you agree that washing your hands with soap is more effective in the removal of pathogens than without soap?		**231**	**96.3**
Q27 Do you agree that washing your hands with alcohol based disinfectant is more effective than with soap?		138	57.5
Q28 Do you agree that the 8 steps for washing hands technique is effective in the removal of pathogens?		**214**	**89.2**
Q29 Do you agree that washing your hands with soap using the 8 steps for washing hands is important?		**207**	**86.3**
Q30 Do you agree that there should be more provisions for alcohol-based disinfectants on public transport systems, such as at the MRT stations or on the buses?		183	76.3
Q31 Do you agree that washing your hands keeps your hands too clean and it may lower your immunity against pathogens?		111	46.3
Q32 Do you agree that the emphasis on hand hygiene should be enforced since kindergarten?		**236**	**98.3**
Q33 Do you agree that you can reduce transmission of disease by covering your mouth when you sneeze?		**234**	**97.5**
Q34 Do you agree that wearing a mask can reduce transmission of disease when you have flu-like symptoms?		**228**	**95.0**
Q35 Do you think an annual hygiene campaign will be effective in encouraging you to have good hand hygiene practice?		**206**	**85.8**
**Food Hygiene - Knowledge and Attitude**			
Q36 Do you agree that good food hygiene can help to reduce diarrheal incidence?		**232**	**96.7**
Q37 Do you agree that it is important to keep fresh food such as vegetable and meat separately in the refrigerator with the appropriate temperature?		**234**	**97.5**
Q38 Do you agree that proper washing of food before cooking can help to reduce the chance of diarrheal incidence?		**235**	**97.9**
Q39 Do you agree that leaving cooked food at room temperature for longer than two hours can increase the chance of diarrheal incidence?		182	75.8
Q40 Do you agree that leftover food should be reheated before serving?		**224**	**93.3**
Q41 Do you agree that checking the expiry date before purchasing or cooking the food can help to reduce the chance of diarrheal incidence?		**233**	**97.1**
Q42 Do you agree that using different chopping boards for meat and vegetables can help to reduce the chance of diarrheal incidence?		181	75.4
Q43 Do you agree that well-cooked food can help to reduce diarrheal incidence?		**237**	**98.8**
Q44 Do you agree that the emphasis of food hygiene should be enforced since kindergarten?		**234**	**97.5**
Q45 Do you think an annual hygiene campaign will be effective to encourage you to have good hygiene practices?		**216**	**90.0**

*Questions for which the response is not indicated would give the values for the positive answer

^Ɨ^Each answer to the sub questions were assigned a value (none = 0, water only = 1, water and soap = 2), the summation of the scores were used in the analysis

With regards to the eight recommended steps of handwashing [28] by the Singapore Health Promotion Board (HPB), 81.3 % agreed hand washing with soap can protect against diarrhoea, and about 89.2 % and 86.3 % agreed that the eight steps of handwashing is effective and important to remove pathogens, respectively. However, only 87.9 % of participants reported following the first step, and subsequently, there was a decreasing trend with only 73.3 %, 63.3 %, 34.2 %, 30.8 %, 26.7 %, 42.9 %, and 75.0 % reported performing the remaining steps 2 to 8, respectively (Table 2). Interestingly, only 11.3 % felt handwashing with soap is troublesome, 86.3 % reported it as a habit for them, and 77.9 % would encourage their family members and friends to pick up the good habit of handwashing with soap.

Although 96.7 % agreed that washing hands with soap can reduce the spread of diseases like HFMD and flu, and 96.3 % agreed that washing hands with soap is more effective than without soap, only 78.8 % would wash their hands with soap more often during disease outbreak (Table 2). Furthermore, even though 92.2 % reported covering their mouth when sneezing, and 97.5 % agreed that covering mouth when sneezing can reduce transmission of disease, only 42.9 % reported washing their hands with soap after sneezing or coughing. Similarly, even though 95 % agreed that wearing a mask can reduce transmission of disease, only 31.3 % would wear a mask in public when sick.

### Food hygiene knowledge, attitude and behaviour

About 96.7 % and 98.3 % of the participants reported keeping fresh fruits and vegetables separately from meat in the refrigerator and ensuring the food was well-cooked before consumption, respectively (Table 2). About 94.2 % checked the expiry date of food packaging before purchasing or cooking the food, and 88.3 % would reheat leftover food before consumption. These behaviours were positively correlated to the high proportion (at least 90 %) of participants who agreed on the importance of performing these actions to reduce diarrhoea. About 49.2 % would still leave cooked food at room temperature for longer than two hours even though 75.8 % agreed that leaving cooked food at room temperature for longer than two hours will increase the chance of diarrhoea (Table 2). Similarly, only 56.7 % had separate chopping boards for vegetables and meat, even though 75.4 % agreed that having two separate chopping boards for meat and vegetables can reduce diarrhoea incidence (Table 2).

Both hawker centres and restaurants were largely chosen based on good recommendations of their food (40.8 % and 62.9 %, respectively) instead of the overall cleanliness of the stall (39.6 %) or the National Environmental Agency (NEA) grading (19.6 % for hawker centres and 37.1 % for restaurant) (Table 2). The NEA grading is a structured system of appraisal for food outlets. It was introduced to motivate licensees to improve and maintain good personal and food hygiene, and housekeeping of their premises [29]. About 58.3 % of the participants reported that they will inform neither NEA nor the restaurant/hawker stall if they experience diarrhoea. Only 19.6 % and 34.6 % would inform NEA/Ministry of Health (MOH) and the restaurant/stall respectively, if they experience diarrhoea after consuming food from the vendors (Table 2). In the event of diarrhoea episode, 37.1 % would prefer to self-medicate and about 49.2 % would prefer to visit a general practitioner (GP) in a private clinic (Table 2).

About 98 % of the participants agreed in early emphasis and training of hand and food hygiene starting from kindergarten (Table 2). More than 85 % also felt an annual hand and food hygiene campaign will be effective to encourage them to sustain good hygiene practices. Moreover, 76.3 % of the participants concurred for provisions of alcohol-based disinfectants to be made available on public transportation systems such as mass rapid trains and buses (Table 2). Furthermore, 63.3 % of the participants felt an online portal will be useful for reporting diarrhoea (Table 2).

### Risk factors of diarrhoea

In order to explore the potential risk factors associated with diarrhoea in the past six-month period, odds ratios were calculated (Table 3; Additional file 1: Table S1). Based on fisher’s exact test, age, marital status and flu were found to be statistically significant. Adjusting for these variables, the adjusted odds ratios (AOR) were calculated for all variables and questions. Being single (AOR = 2.29; 95 % CI = 1.16-4.48) and having flu in the past six month period (AOR = 3.24; 95 % CI = 1.74-6.06) were risk factors of having diarrhoea in the past six months. Participants who preferred self-medication were twice more likely to experience diarrhoea (AOR = 2.07, 95 % CI = 1.06–4.12) compared to those who preferred to consult a GP. Participants who washed their hands with water before attending to children or sick persons (Q4H) were three times less likely to experience diarrhoea as compared to participants who do not washed their hands (AOR = 0.30; 95 % CI = 0.11–0.82). Furthermore, participants who washed their hands with water (AOR = 0.16, 95 % CI = 0.05–0.45) as well as water with soap (AOR = 0.29; 95 % CI = 0.12–0.72) after attending to children or sick persons (Q4I) had three times lower risk of diarrhoea incidence as compared to participants who do not washed their hands at all (Table 3).

**Table 3:** Significant risk factors of having diarrhoea

	Diarrhoea^**#**^												
Variables	None N = 180	%	Yes N = 60	%	p-value^	Crude OR	p-value	95 % CI Range		Adjusted OR^**+**^	p-value	95 % CI Range	
**Median Age**	43		36		**0.0188**	**0.98**	**0.0204**	0.96	1.00	0.99	0.4421	0.97	1.01
(Interquartile Range)	(33–55)		(29–45.25)										
**Marital Status**													
Married	145	80.6	39	65.0		1.00				1.00			
Single	35	19.4	21	35.0	**0.0211**	**2.23**	**0.0150**	**1.16**	**4.25**	**2.29**	**0.0154**	**1.16**	**4.48**
**Ethnic Group**													
Chinese	117	65.0	42	70.0		1.00				1.00			
Malay	19	10.6	11	18.3		1.61	0.2544	0.69	3.63	1.53	0.3276	0.64	3.58
Indian	29	16.1	3	5.0		**0.29**	**0.0492**	**0.07**	**0.87**	0.31	0.0727	0.07	0.98
Others	15	8.3	4	6.7	0.0711	0.74	0.6149	0.20	2.18	0.69	0.5509	0.18	2.15
**Flu** ^**#**^													
No	137	76.1	30	50.0		1.00				1.00			
Yes	43	23.9	30	50.0	**0.0003**	**3.19**	**0.0002**	**1.73**	**5.90**	**3.24**	**0.0002**	**1.74**	**6.06**
**Q4H**													
None	20	11.1	12	20.0		1.00				1.00			
Water only	62	34.4	12	20.0		**0.32**	**0.0190**	**0.12**	**0.83**	**0.30**	**0.0194**	**0.11**	**0.82**
Water and Soap	98	54.4	36	60.0	**0.0495**	0.61	0.2359	0.27	1.41	0.56	0.1917	0.24	1.36
**Q4I**													
None	12	6.7	14	23.3		1.00				1.00			
Water only	57	31.7	10	16.7		**0.15**	**0.0003**	**0.05**	**0.41**	**0.16**	**0.0008**	**0.05**	**0.45**
Water and Soap	111	61.7	36	60.0	**0.0009**	**0.28**	**0.0034**	**0.12**	**0.65**	**0.29**	**0.0077**	**0.12**	**0.72**
**Q4M**													
<30 sec	89	49.4	40	66.7		1.00				1.00			
30 sec – 60 sec	63	35.0	12	20.0		**0.42**	**0.0197**	**0.20**	**0.85**	**0.44**	**0.0302**	**0.20**	**0.90**
>60 sec	28	15.6	8	13.3	0.0543	0.64	0.3074	0.25	1.46	0.81	0.6472	0.31	1.95
**Q16**													
No	0	0.0	4	6.7		1.00				1.00			
Yes	180	100.0	56	93.3	**0.0036**	**0.01**	**<0.0001**	**0.00**	**6487.54**	**0.01**	**<0.0001**	**0.00**	**2979.95**
Q20*													
0	106	58.9	45	75.0		1.00				1.00			
1	74	41.1	15	25.0	0.0305	**0.48**	**0.0271**	**0.24**	**0.90**	0.52	0.0606	0.26	1.01
Q21*													
0	99	55.0	41	68.3		1.00				1.00			
1	54	30.0	16	26.7		0.72	0.3245	0.36	1.37	0.69	0.2974	0.33	1.37
2	27	15.0	3	5.0	0.0682	**0.27**	**0.0387**	**0.06**	**0.81**	**0.26**	**0.0393**	**0.06**	**0.82**
**Q23**													
Visit a GP	95	52.8	23	38.3		1.00				1.00			
Self-medicate	61	33.9	28	46.7		**1.90**	**0.0496**	**1.00**	**3.62**	**2.07**	**0.0346**	**1.06**	**4.12**
Visit Hospital	6	3.3	1	1.7		0.69	0.7354	0.04	4.31	0.45	0.4796	0.02	3.03
Visit Polyclinic	18	10.0	8	13.3	0.1836	1.84	0.2098	0.68	4.65	1.81	0.2570	0.63	4.94

CI = Confidence interval, OR = Odds ratio

^- Fisher Exact’s Test

*Each variable in these questions were given a score. Question 20 follows the same scoring as the previous question. Question 21: a score of 1 was given for each method of giving feedback

+ Age, marital status and having flu over the last 6 months were used to calculate the adjusted OR in the multivariate logistic regression model

# Participants who self-reported having at least one episode of diarrhoea or flu, respectively, in the past six months

The longer duration (between 30 s to a minute) of hand washing (Q4M) reduced the risk of diarrhoea by about two times (AOR = 0.44; 95 % CI = 0.20-0.90; Table 3) as compared to participants who washed their hands for less than 30 s. In addition, there were significantly high risk of diarrhoea when individuals reported not ensuring the food is well-cooked before consumption (P < 0.00001) (Q16; Table 3). Finally, participants who preferred to provide feedback to NEA/MOH and the restaurant/hawker stall when they experience diarrhoea (Q21) were about four times less likely (AOR = 0.26; 95 % CI = 0.06-0.82) to have diarrhoea as compared to those who preferred not to provide feedback at all (Table 3).

## Discussion

Our results showed that there was good knowledge and attitude towards hand and food hygiene among the residents in the heartland of a developed Singapore. The vast majority of participants were well-informed of the recommended hand and food hygiene behaviour and practices to minimise risk of diarrhoea disease. About 77 % of the expected knowledge and attitude were observed in at least 80 % of the participants. This was likely due to the national hygiene campaigns launched through HPB since 2001, which had been effective in raising awareness and knowledge of simple hygiene practices, similarly in other countries [15–17, 30–32]. Nonetheless, the good knowledge and attitude did not translate and sustain positively into good behaviour and practice of hygiene in their daily lives. There were only about 31 % of the expected behaviours and practises observed in at least 80 % of the participants. These may be due to a few key reasons. First, the busy working lifestyle, among adults range from 32–52, and of median age 36 who were at higher risk of diarrhoea, may have made food and hand hygiene the least concern over time, especially when low prevalence of severe and fatal diarrhoea were reported in a developed community. However, further qualitative study would be required to fully understand the association behind this observation. Second, the high standard of medical, water and sanitation facilities may have also created a false sense of health security, which may have resulted in complacency and increased redundancy in practising good hygiene. Third, participants may have the incorrect assumption that severe diarrhoea disease would only affect children, but not adults since adults were presumed to have a stronger immune system than children [2]. Lastly, there may still be a lack of infrastructure or effective programmes to promote sustainable good hygiene behaviour in households. These deserve further studies and assessments.

Our results suggested complacency among participants as they believed their current behaviour and practices were sufficient to prevent diarrhoea diseases. Although most participants reported washing hands with soap more than three times a day and was not troublesome, the response for routinely performing all the recommended eight steps of handwashing was still very low. Of concern, the behaviour of handwashing before and after contact with sick individuals and children were considered crucial to breaking transmission of diseases, as according to the WHO “My 5 moments of hand hygiene” [4, 30, 31], but there were only less than 65 % of the participants that practised these. This further highlighted a substantial gap between the behaviour of the community and the public health official’s perception on proper hand hygiene and cleanliness.

Remarkably, NEA grading had the least influence on the choices of food outlets on the participants, as compared to recommendation of good food. This is likely due to the fact that the NEA grading is only assessed once a year, which the participants may find it inaccurate to assess the level of cleanliness at any particular time. The general public may also have a perception that the authority would have already closed down stalls which are evaluated as unhygienic, and stalls that are opened should be considered sufficiently hygienic. The low proportion of people who are likely to inform NEA/MOH of diarrhoea disease clearly suggested underreporting of diarrhoea incidence, and hence, more effort is required to improve the surveillance of diarrhoea diseases. One possible alternative is an online platform or application that could be more convenient for the public to self-report while enabling confidentiality, and 63.3 % supported this intervention. The online self-report portal would also be very useful to capture the potential 37.1 % of the participants that is likely missing in the national surveillance system who prefer to self-medicate when they had diarrhoea as shown in other studies [33–35].

Proactive community partnership and engagement such as having regular community health ambassadors advocating good hygiene messages with soap/hand sanitizer using a door-to-door approach, and having open dialogue sessions may help to improve the compliance to good hygiene practices [36]. These ambassadors can be the local trained residents, or students with guidance from a professional staff from a social organisations such as the HPB, NEA, People’s Association [37] and the Public Hygiene Council [38]. A study in a Singapore polyclinic had also shown that by adopting an open communication platform between nurses and infection control team on compliance identification and effective solutions, there was improvements in hand hygiene compliance. However, the long term sustainability remained to be assessed [30].

Almost all participants were highly supportive for children to learn and practise these good hygiene habits during early childhood education period, which has also been shown to be effective in reducing diarrhoea and absenteeism [39]. It was reported that the school hygiene promotion, water treatment interventions and school sanitation improvements were not as effective to reduce diarrhoea in schools with greater water availability, as compared to in schools with very poor water availability [40, 41]. In addition, an annual hand and food hygiene campaign, which had been shown to be effective in reducing diarrhoea disease even in mostly rural areas [13, 14, 42]. These highlighted the importance of sustainable compliance to good hygiene behaviour, beside good knowledge and infrastructure. However, the local campaigns should be strongly tailored towards educating the working adults in Singapore, on the current gaps to adopt the complete good hygiene practices with closer monitoring. Moreover, this would also require regular evaluation to obtain the most effective method of delivering the hygiene education [43].

Participants were also supportive of having alcohol-based hand sanitizers conveniently located within public transport systems, where it may potentially be useful to prevent and delay transmission of diseases, particularly during an epidemic, and this may also address some of the barriers to better hand hygiene compliance [30]. However, further study is required to assess the efficacy of providing hand sanitizer in public transport to reduce diarrhoea, as the use of public transport was reported to be less likely to spread diseases like influenza infection compared to in the home environment. In addition, handwashing as well as the use of alcohol-based hand sanitizers were reported to have minimal protective effect against diseases such as influenza infection [44].

The behaviour of washing hands before and after interaction with children and sick people, which were highly recommended practises for infection control, had a large protective effect against having diarrhoea. This reemphasized the importance of this simple public health practice that the community can perform to reduce diarrhoea, and also potentially stop transmission of other infectious diseases [45, 46]. Not surprisingly, the duration of washing hands can also help to reduce diarrhoea, probably as a surrogate for the thoroughness of hand washing, similarly observed in other published studies [31, 47, 48]. We postulated that the increased risk of diarrhoea among singles and those who had flu in the past six months is probably due to lower family and social motivation for compliance of good hygiene behaviour, compared to married individuals with children under the age of five. Moreover, we are proposing that having flu in the past six months is likely to be a social predictor of having diarrhoea around the same period as a result of poor hand hygiene behaviours, instead of a clinical predictor of flu severity. This is also supported by the fact that the association of diarrhoea as a clinical symptom of flu is less likely to be associated with young adults infected with influenza [49, 50], compared with children hospitalised with influenza [51, 52]. Furthermore, one study had shown the potential impact of good hand hygiene behaviour advocated during influenza pandemic can help to reduce acute diarrhoea during the same period [53]. Moreover, the higher risk of diarrhoea among individuals who preferred self-medication over those who would visit the general practitioner (GP), is likely due to their complacent attitude of good hygiene and the mild disease, which does not require a higher standard of medical care. On the contrary, participants who preferred to provide feedback to NEA/MOH, hawker stalls and restaurants were more likely to be concerned on the impact of diarrhoea disease on their health, and hence, may have influenced their actions that could potentially reduce diarrhoea.

This study has several limitations. First, the cross-sectional nature of this study does not allowed the temporal relationship to be established between the explanatory and outcome variable. Hence, there may be Hawthorne effect. Second, we were unable to perform randomised sampling of other constituencies in Singapore, and to do sampling from every zone and block in each constituency which would increase the validity and accuracy of the survey’s findings. Some of the zones sampled tend to have newer blocks, where the demographics and housing type may be invariably different. However, this bias should be minimal as the proportion of ethnic groups would be similar across other developed communities in Singapore since there is a housing policy to sustain a well-balanced ethnic groups in all HDB blocks [54]. Third, the small percentage of respondents and the lack of the housing demographics among those who rejected the study, may limit the generalisability of results to the general population in Singapore. However, the guiding reference from HDB [26] had shown that our subpopulation is very similar to the general housing demographics in Singapore, and so the variability of impact may be minimal. Furthermore, there is at least 79 % power with the current sample size to detect true positive associations with risk effect of 1.8 and proportion difference of 20 % with 5 % level of significance. Fourth, as this survey is conducted in English, some willing participants may reject to participate due to a lack of comprehension of the survey questions. However, this number is small as most residents know English, and Chinese translated version of the survey was also made available. Fourth, we were not able to assess for non-respondent bias as the majority of non-responders were unwilling to provide the minimal demographic information for analysis. Fifth, the choices provided were categorical, which may limit the possibilities of answers as compared to free-text design. However, categorical questions can provide simple and direct answers, and the consistency required for the data collection process and the subsequent analysis. Lastly, recall bias may be present in the survey, but to a minimal extent. This is because these questions were generally crafted to target their daily general practise and attitude. Furthermore, the self-reporting of at least one episode of diarrhoea or flu over the past six month minimises misclassification bias as diarrhoea and flu episode can be an eventful one. Nevertheless, this is thus limited in the fact that we cannot assume that the participants had both flu and diarrhoea during the same time. Additionally, there should be minimal response bias towards the expected hygiene practices because the behaviour questions were asked before the knowledge and attitude questions.

## Conclusions

Diarrhoea disease may not have resulted in as much public health burden as compared to chronic diseases such as diabetes, but it has potential of incurring significant economic impact in the near future, if not managed appropriately, like in many other developing and developed countries [55–64]. Therefore, prevention of diarrhoea through having good hygiene practices is important to sustain in the developed residential community. From this study, we found a high level of knowledge and attitude on hand and food hygiene amongst the participants in a developed residential community. However, the hygiene practice and behaviour were not as highly complied as expected. Future interventions should focus more on advocating sustainable behaviour of good hygiene, particularly among working adults, and to highlight the risk factors of diarrhoea. This may be achieved with more active community partnership and engagements driven by the relevant social organisations. The development of evidence-based surveillance capabilities, policies, and preventive measures to increase good hygiene compliance will be critical to reduce diarrhoea in a developed residential community.

## Additional file

Additional file 1:
**Demographics and question odds ratio analysis between participants with and without diarrhoea.**

## Abbreviations

HDB: Housing development board
HPB: Health promotion board
NEA: National environmental Agency
MOH: Ministry of health
