# Supplementary material for: Current knowledge, attitude and behaviour of hand and food hygiene in a developed residential community of Singapore: a cross-sectional survey
Source: BMC Public Health. 2015 Jun 21;15:577. doi: 10.1186/s12889-015-1910-3 (PMC4475322; doi:10.1186/s12889-015-1910-3)
Supplement: Additional file 1: — Demographics and question odds ratio analysis between participants with and without diarrhoea. [file 12889_2015_1910_MOESM1_ESM.docx]

**Supplementary Table 3: Demographics and question odds ratio analysis between participants with and without diarrhoea**

|  | **Diarrhoea Frequency** | | | |  |  |  |  |  |  |  |  |  |
| --- | --- | --- | --- | --- | --- | --- | --- | --- | --- | --- | --- | --- | --- |
| **Variables** | **None**  **N=180** | **%** | **At least 1**  **N=60** | **%** | **Fisher exact test**  **p-value*** | **Crude**  **OR** | **p-value** | **95% CI Range** | | **Adjusted**  **OR^+^** | **p-value** | **95% CI Range** | |
| **Median Age** | 43 |  | 36 |  | 0.0188 | **0.98** | **0.0204** | 0.96 | 1.00 | 0.99 | 0.4421 | 0.97 | 1.01 |
| (Interquartile Range) | (33-55) |  | (29-45.25) |  |  |  |  |  |  |  |  |  |  |
| **Gender** |  |  |  |  |  |  |  |  |  |  |  |  |  |
| Female | 95 | 52.8 | 27 | 45.0 |  | 1.00 |  |  |  | 1.00 |  |  |  |
| Male | 85 | 47.2 | 33 | 55.0 | 0.3019 | 1.37 | 0.2975 | 0.76 | 2.47 | 1.39 | 0.3017 | 0.75 | 2.59 |
| **Marital Status** |  |  |  |  |  |  |  |  |  |  |  |  |  |
| Married | 145 | 80.6 | 39 | 65.0 |  | 1.00 |  |  |  | 1.00 |  |  |  |
| Single | 35 | 19.4 | 21 | 35.0 | 0.0211 | **2.23** | **0.0150** | **1.16** | **4.25** | **2.29** | **0.0154** | **1.16** | **4.48** |
| **Ethnic Group** |  |  |  |  |  |  |  |  |  |  |  |  |  |
| Chinese | 117 | 65.0 | 42 | 70.0 |  | 1.00 |  |  |  | 1.00 |  |  |  |
| Malay | 19 | 10.6 | 11 | 18.3 |  | 1.61 | 0.2544 | 0.69 | 3.63 | 1.53 | 0.3276 | 0.64 | 3.58 |
| Indian | 29 | 16.1 | 3 | 5.0 |  | **0.29** | **0.0492** | **0.07** | **0.87** | 0.31 | 0.0727 | 0.07 | 0.98 |
| Others | 15 | 8.3 | 4 | 6.7 | 0.0711 | 0.74 | 0.6149 | 0.20 | 2.18 | 0.69 | 0.5509 | 0.18 | 2.15 |
| **Citizenship** |  |  |  |  |  |  |  |  |  |  |  |  |  |
| Singaporean | 136 | 75.6 | 46 | 76.7 |  | 1.00 |  |  |  | 1.00 |  |  |  |
| Permanent Resident | 27 | 15.0 | 12 | 20.0 |  | 1.31 | 0.4800 | 0.60 | 2.76 | 1.09 | 0.8336 | 0.48 | 2.38 |
| Work Permit | 17 | 9.4 | 2 | 3.3 | 0.2493 | 0.35 | 0.1684 | 0.05 | 1.28 | 0.34 | 0.1721 | 0.05 | 1.32 |
| **Citizenship** |  |  |  |  |  |  |  |  |  |  |  |  |  |
| Singaporean | 136 | 75.6 | 46 | 76.7 |  | 1.00 |  |  |  | 1.00 |  |  |  |
| Non-Singaporean | 44 | 24.4 | 14 | 23.3 | 1.0000 | 0.94 | 0.8618 | 0.46 | 1.84 | 0.82 | 0.5842 | 0.39 | 1.65 |
| **Education** |  |  |  |  |  |  |  |  |  |  |  |  |  |
| Pre-Secondary | 94 | 52.2 | 26 | 43.3 |  | 1.00 |  |  |  | 1.00 |  |  |  |
| Post-Secondary | 86 | 47.8 | 34 | 56.7 | 0.2967 | 1.43 | 0.2341 | 0.80 | 2.59 | 1.05 | 0.8747 | 0.56 | 1.98 |
| **Occupational Status** |  |  |  |  |  |  |  |  |  |  |  |  |  |
| Employed | 115 | 63.9 | 38 | 63.3 |  | 1.00 |  |  |  | 1.00 |  |  |  |
| Housewife | 28 | 15.6 | 9 | 15.0 |  | 0.97 | 0.9483 | 0.40 | 2.18 | 1.40 | 0.4622 | 0.55 | 3.39 |
| Healthcare worker | 2 | 1.1 | 3 | 5.0 |  | 4.54 | 0.1045 | 0.73 | 35.44 | 6.27 | 0.0602 | 0.92 | 52.36 |
| Unemployed | 25 | 13.9 | 6 | 10.0 |  | 0.73 | 0.5154 | 0.25 | 1.80 | 0.84 | 0.7280 | 0.28 | 2.20 |
| Student | 10 | 5.6 | 4 | 6.7 | 0.4323 | 1.21 | 0.7582 | 0.32 | 3.85 | 0.56 | 0.4056 | 0.13 | 2.09 |
| **Occupational Status** |  |  |  |  |  |  |  |  |  |  |  |  |  |
| Employed | 117 | 65.0 | 41 | 68.3 |  | 1.00 |  |  |  | 1.00 |  |  |  |
| Unemployed | 63 | 35.0 | 19 | 31.7 | 0.7535 | 0.86 | 0.6370 | 0.45 | 1.59 | 0.90 | 0.7638 | 0.46 | 1.73 |
| **Number of residing members** |  |  |  |  |  |  |  |  |  |  |  |  |  |
| 4 | 50 | 27.8 | 18 | 30.0 |  | 1.00 |  |  |  | 1.00 |  |  |  |
| <3 | 28 | 15.6 | 9 | 15.0 |  | 0.89 | 0.8101 | 0.34 | 2.21 | 0.99 | 0.9874 | 0.36 | 2.60 |
| 3 | 27 | 15.0 | 8 | 13.3 |  | 0.82 | 0.6895 | 0.30 | 2.09 | 0.82 | 0.6916 | 0.29 | 2.16 |
| 5 | 31 | 17.2 | 11 | 18.3 |  | 0.99 | 0.9742 | 0.40 | 2.34 | 1.01 | 0.9808 | 0.40 | 2.51 |
| >5 | 44 | 24.4 | 14 | 23.3 | 0.9952 | 0.88 | 0.7644 | 0.39 | 1.98 | 0.84 | 0.6825 | 0.35 | 1.95 |
| **Number of residing members** |  |  |  |  |  |  |  |  |  |  |  |  |  |
| <3 | 28 | 15.6 | 9 | 15.0 |  | 1.00 |  |  |  |  |  |  |  |
| 3--5 | 108 | 60.0 | 37 | 61.7 |  | 1.07 | 0.8815 | 0.47 | 2.58 | 0.96 | 0.9371 | 0.41 | 2.44 |
| >5 | 43 | 23.9 | 14 | 23.3 | 1.0000 | 1.01 | 0.9792 | 0.39 | 2.73 | 0.86 | 0.7684 | 0.31 | 2.43 |
| **Children =<5 Years** |  |  |  |  |  |  |  |  |  |  |  |  |  |
| Yes | 137 | 76.1 | 48 | 80.0 |  | 1.00 |  |  |  | 1.00 |  |  |  |
| No | 43 | 23.9 | 12 | 20.0 | 0.5980 | 0.80 | 0.5354 | 0.37 | 1.60 | 0.78 | 0.5466 | 0.34 | 1.69 |
| **Residences** |  |  |  |  |  |  |  |  |  |  |  |  |  |
| 4 Room | 102 | 56.7 | 31 | 51.7 |  | 1.00 |  |  |  | 1.00 |  |  |  |
| 2-3 Room | 20 | 11.1 | 9 | 15.0 |  | 1.48 | 0.3839 | 0.59 | 3.51 | 1.45 | 0.4295 | 0.56 | 3.59 |
| 5 Room & Executive | 58 | 32.2 | 20 | 33.3 | 0.6529 | 1.13 | 0.7025 | 0.59 | 2.16 | 0.96 | 0.9182 | 0.48 | 1.91 |
| **Comorbidities** |  |  |  |  |  |  |  |  |  |  |  |  |  |
| **Diabetes** |  |  |  |  |  |  |  |  |  |  |  |  |  |
| No | 171 | 95.0 | 58 | 96.7 |  | 1.00 |  |  |  | 1.00 |  |  |  |
| Yes | 29 | 16.1 | 2 | 3.3 | 0.7356 | 0.66 | 0.5954 | 0.10 | 2.63 | 0.84 | 0.8310 | 0.12 | 3.60 |
| **Hypertension** |  |  |  |  |  |  |  |  |  |  |  |  |  |
| No | 151 | 83.9 | 55 | 91.7 |  | 1.00 |  |  |  | 1.00 |  |  |  |
| Yes | 29 | 16.1 | 5 | 8.3 | 0.1982 | 0.47 | 0.1419 | 0.16 | 1.19 | 0.61 | 0.3421 | 0.19 | 1.59 |
| **Asthma** |  |  |  |  |  |  |  |  |  |  |  |  |  |
| No | 176 | 97.8 | 60 | 100.0 |  |  |  |  |  | 1.00 |  |  |  |
| Yes | 4 | 2.2 | 0 | 0.0 | 0.5746 | 0.03 | 1.0000 | 0.00 | 99.15 | 0.42 | 1.0000 | 0.15 | 1.45 |
| **Allergy** |  |  |  |  |  |  |  |  |  |  |  |  |  |
| No | 174 | 96.7 | 57 | 95.0 |  | 1.00 |  |  |  | 1.00 |  |  |  |
| Yes | 6 | 3.3 | 3 | 5.0 | 0.6945 | 0.56 | 0.5589 | 0.31 | 5.98 | 1.24 | 0.7812 | 0.23 | 5.40 |
| **Smoking Status** |  |  |  |  |  |  |  |  |  |  |  |  |  |
| Non-smoker | 145 | 80.6 | 47 | 78.3 |  | 1.00 |  |  |  | 1.00 |  |  |  |
| Smoker | 19 | 10.6 | 11 | 18.3 |  | 1.79 | 0.1616 | 0.77 | 3.97 | 1.67 | 0.2406 | 0.69 | 3.88 |
| Ex-smoker | 16 | 8.9 | 2 | 3.3 | 0.1528 | 0.39 | 0.2150 | 0.06 | 1.42 | 0.36 | 0.2046 | 0.05 | 1.43 |
| **Smoking Status** |  |  |  |  |  |  |  |  |  |  |  |  |  |
| Non-smoker | 145 | 80.6 | 47 | 78.3 |  | 1.00 |  |  |  | 1.00 |  |  |  |
| Smoker | 35 | 19.4 | 13 | 21.7 | 0.7119 | 1.15 | 0.7095 | 0.54 | 2.30 | 1.09 | 0.8324 | 0.50 | 2.27 |
| **Flu Incidence** |  |  |  |  |  |  |  |  |  |  |  |  |  |
| No | 137 | 76.1 | 30 | 50.0 |  | 1.00 |  |  |  | 1.00 |  |  |  |
| Yes | 43 | 23.9 | 30 | 50.0 | 0.0003 | **3.19** | **0.0002** | **1.73** | **5.90** | **3.24** | **0.0002** | **1.74** | **6.06** |
| **Q1** |  |  |  |  |  |  |  |  |  |  |  |  |  |
| 0-8 times | 95 | 52.8 | 29 | 48.3 |  | 1.00 |  |  |  | 1.00 |  |  |  |
| >9 times | 85 | 47.2 | 31 | 51.7 | 0.5552 | 1.19 | 0.5510 | 0.67 | 2.15 | 1.33 | 0.3688 | 0.72 | 2.48 |
| **Q2** |  |  |  |  |  |  |  |  |  |  |  |  |  |
| 0-8 times | 135 | 75.0 | 44 | 73.3 |  | 1.00 |  |  |  | 1.00 |  |  |  |
| >9 times | 45 | 25.0 | 16 | 26.7 | 0.8643 | 1.09 | 0.7974 | 0.55 | 2.09 | 1.26 | 0.5218 | 0.62 | 2.49 |
| **Q3** |  |  |  |  |  |  |  |  |  |  |  |  |  |
| Never | 147 | 81.7 | 45 | 75.0 |  | 1.00 |  |  |  |  |  |  |  |
| 1-3 times | 24 | 13.3 | 9 | 15.0 |  | 1.23 | 0.6341 | 0.51 | 2.75 | 1.09 | 0.8505 | 0.43 | 2.54 |
| >=4 times | 9 | 5.0 | 6 | 10.0 | 0.3194 | 2.18 | 0.1600 | 0.70 | 6.37 | 2.02 | 0.2307 | 0.61 | 6.29 |
| **Q4A** |  |  |  |  |  |  |  |  |  |  |  |  |  |
| None | 4 | 2.2 | 1 | 1.7 |  | 1.00 |  |  |  | 1.00 |  |  |  |
| Water only | 18 | 10.0 | 6 | 10.0 |  | 1.33 | 0.8126 | 0.15 | 28.84 | 0.79 | 0.8472 | 0.09 | 17.33 |
| Water and Soap | 158 | 87.8 | 53 | 88.3 | 1.0000 | 1.34 | 0.7946 | 0.19 | 26.56 | 0.67 | 0.7300 | 0.09 | 13.55 |
| **Q4B** |  |  |  |  |  |  |  |  |  |  |  |  |  |
| None | 10 | 5.6 | 3 | 5.0 |  | 1.00 |  |  |  | 1.00 |  |  |  |
| Water only | 83 | 46.1 | 27 | 45.0 |  | 1.08 | 0.9072 | 0.31 | 5.09 | 1.26 | 0.7447 | 0.34 | 6.07 |
| Water and Soap | 87 | 48.3 | 30 | 50.0 | 0.9688 | 1.15 | 0.8404 | 0.33 | 5.38 | 1.38 | 0.6497 | 0.38 | 6.63 |
| **Q4C** |  |  |  |  |  |  |  |  |  |  |  |  |  |
| None | 7 | 3.9 | 2 | 3.3 |  | 1.00 |  |  |  | 1.00 |  |  |  |
| Water only | 66 | 36.7 | 24 | 40.0 |  | 1.27 | 0.7731 | 0.28 | 8.94 | 1.30 | 0.7599 | 0.27 | 9.47 |
| Water and Soap | 107 | 59.4 | 34 | 56.7 | 0.9254 | 1.11 | 0.8976 | 0.25 | 7.70 | 1.20 | 0.8267 | 0.26 | 8.64 |
| **Q4D** |  |  |  |  |  |  |  |  |  |  |  |  |  |
| None | 7 | 3.9 | 2 | 3.3 |  | 1.00 |  |  |  | 1.00 |  |  |  |
| Water only | 43 | 23.9 | 9 | 15.0 |  | 0.73 | 0.7241 | 0.15 | 5.46 | 0.63 | 0.6222 | 0.12 | 5.01 |
| Water and Soap | 130 | 72.2 | 49 | 81.7 | 0.3402 | 1.32 | 0.7352 | 0.31 | 9.06 | 1.30 | 0.7563 | 0.28 | 9.38 |
| **Q4E** |  |  |  |  |  |  |  |  |  |  |  |  |  |
| None | 1 | 0.6 | 0 | 0.0 |  |  |  |  |  |  |  |  |  |
| Water only | 22 | 12.2 | 10 | 16.7 |  | 1.00 |  |  |  | 1.00 |  |  |  |
| Water and Soap | 157 | 87.2 | 50 | 83.3 | 0.5404 | 0.73 | 0.4500 | 0.33 | 1.71 | 0.68 | 0.3745 | 0.30 | 1.64 |
| **Q4F** |  |  |  |  |  |  |  |  |  |  |  |  |  |
| None | 0 | 0.0 | 0 | 0.0 |  |  |  |  |  |  |  |  |  |
| Water only | 50 | 27.8 | 16 | 26.7 |  | 1.00 |  |  |  | 1.00 |  |  |  |
| Water and Soap | 130 | 72.2 | 44 | 73.3 | 1.0000 | 1.06 | 0.8674 | 0.56 | 2.09 | 0.82 | 0.5845 | 0.41 | 1.68 |
| **Q4G** |  |  |  |  |  |  |  |  |  |  |  |  |  |
| None | 10 | 5.6 | 2 | 3.3 |  | 1.00 |  |  |  | 1.00 |  |  |  |
| Water only | 38 | 21.1 | 14 | 23.3 |  | 1.84 | 0.4646 | 0.42 | 12.96 | 1.87 | 0.4639 | 0.40 | 13.52 |
| Water and Soap | 132 | 73.3 | 44 | 73.3 | 0.8565 | 1.67 | 0.5200 | 0.42 | 11.11 | 1.53 | 0.5970 | 0.37 | 10.44 |
| **Q4H** |  |  |  |  |  |  |  |  |  |  |  |  |  |
| None | 20 | 11.1 | 12 | 20.0 |  | 1.00 |  |  |  | 1.00 |  |  |  |
| Water only | 62 | 34.4 | 12 | 20.0 |  | **0.32** | **0.0190** | **0.12** | **0.83** | **0.30** | **0.0194** | **0.11** | **0.82** |
| Water and Soap | 98 | 54.4 | 36 | 60.0 | 0.0495 | 0.61 | 0.2359 | 0.27 | 1.41 | 0.56 | 0.1917 | 0.24 | 1.36 |
| **Q4I** |  |  |  |  |  |  |  |  |  |  |  |  |  |
| None | 12 | 6.7 | 14 | 23.3 |  | 1.00 |  |  |  | 1.00 |  |  |  |
| Water only | 57 | 31.7 | 10 | 16.7 |  | **0.15** | **0.0003** | **0.05** | **0.41** | **0.16** | **0.0008** | **0.05** | **0.45** |
| Water and Soap | 111 | 61.7 | 36 | 60.0 | 0.0009 | **0.28** | **0.0034** | **0.12** | **0.65** | **0.29** | **0.0077** | **0.12** | **0.72** |
| **Q4J** |  |  |  |  |  |  |  |  |  |  |  |  |  |
| None | 39 | 21.7 | 10 | 16.7 |  | 1.00 |  |  |  | 1.00 |  |  |  |
| Water only | 63 | 35.0 | 25 | 41.7 |  | 1.55 | 0.3053 | 0.69 | 3.70 | 1.31 | 0.5376 | 0.56 | 3.24 |
| Water and Soap | 78 | 43.3 | 25 | 41.7 | 0.5904 | 1.25 | 0.5974 | 0.56 | 2.97 | 1.10 | 0.8252 | 0.48 | 2.68 |
| **Q4K** |  |  |  |  |  |  |  |  |  |  |  |  |  |
| None | 10 | 5.6 | 8 | 13.3 |  | 1.00 |  |  |  | 1.00 |  |  |  |
| Water only | 33 | 18.3 | 13 | 21.7 |  | 0.49 | 0.2191 | 0.16 | 1.54 | 0.50 | 0.2531 | 0.15 | 1.67 |
| Water and Soap | 135 | 75.0 | 37 | 61.7 | 0.0792 | 0.34 | 0.0355 | 0.13 | 0.95 | 0.35 | 0.0510 | 0.12 | 1.03 |
| **Q4L** |  |  |  |  |  |  |  |  |  |  |  |  |  |
| Water | 14 | 7.8 | 5 | 8.3 |  | 1.00 |  |  |  | 1.00 |  |  |  |
| Soap Bar | 21 | 11.7 | 4 | 6.7 |  | 0.53 | 0.4047 | 0.11 | 2.35 | 0.46 | 0.3223 | 0.09 | 2.14 |
| Liquid Soap | 145 | 80.6 | 51 | 85.0 | 0.5766 | 0.98 | 0.9777 | 0.36 | 3.17 | 0.82 | 0.7197 | 0.28 | 2.70 |
| **Q4M** |  |  |  |  |  |  |  |  |  |  |  |  |  |
| <30 sec | 89 | 49.4 | 40 | 66.7 |  | 1.00 |  |  |  | 1.00 |  |  |  |
| 30 sec – 60 sec | 63 | 35.0 | 12 | 20.0 |  | 0.42 | 0.0197 | 0.20 | 0.85 | 0.44 | 0.0302 | 0.20 | 0.90 |
| >60 sec | 28 | 15.6 | 8 | 13.3 | 0.0543 | 0.64 | 0.3074 | 0.25 | 1.46 | 0.81 | 0.6472 | 0.31 | 1.95 |
| **Q4SUM*** | 21 |  | 21 |  | 0.4501 | 0.97 | 0.4487 | 0.91 | 1.05 | 0.97 | 0.4142 | 0.90 | 1.05 |
| (Interquartile Range) | (17-23) |  | (17.75-23) |  |  |  |  |  |  |  |  |  |  |
| **Q5A** |  |  |  |  |  |  |  |  |  |  |  |  |  |
| No | 23 | 12.8 | 6 | 10.0 |  | 1.00 |  |  |  | 1.00 |  |  |  |
| Yes | 157 | 87.2 | 54 | 90.0 | 0.6535 | 1.32 | 0.5685 | 0.54 | 3.72 | 0.96 | 0.9406 | 0.37 | 2.81 |
| **Q5B** |  |  |  |  |  |  |  |  |  |  |  |  |  |
| No | 47 | 26.1 | 17 | 28.3 |  | 1.00 |  |  |  | 1.00 |  |  |  |
| Yes | 133 | 73.9 | 43 | 71.7 | 0.7383 | 0.89 | 0.7361 | 0.47 | 1.75 | 0.88 | 0.7258 | 0.45 | 1.79 |
| **Q5C** |  |  |  |  |  |  |  |  |  |  |  |  |  |
| No | 65 | 36.1 | 23 | 38.3 |  | 1.00 |  |  |  | 1.00 |  |  |  |
| Yes | 115 | 63.9 | 37 | 61.7 | 0.7593 | 0.91 | 0.7571 | 0.50 | 1.68 | 0.96 | 0.9075 | 0.51 | 1.83 |
| **Q5D** |  |  |  |  |  |  |  |  |  |  |  |  |  |
| No | 123 | 68.3 | 35 | 58.3 |  | 1.00 |  |  |  | 1.00 |  |  |  |
| Yes | 57 | 31.7 | 25 | 41.7 | 0.1613 | 1.54 | 0.1587 | 0.84 | 2.81 | 1.76 | 0.0825 | 0.93 | 3.33 |
| **Q5E** |  |  |  |  |  |  |  |  |  |  |  |  |  |
| No | 125 | 69.4 | 41 | 68.3 |  | 1.00 |  |  |  | 1.00 |  |  |  |
| Yes | 55 | 30.6 | 19 | 31.7 | 0.8729 | 1.05 | 0.8718 | 0.55 | 1.96 | 1.19 | 0.6012 | 0.61 | 2.31 |
| **Q5F** |  |  |  |  |  |  |  |  |  |  |  |  |  |
| No | 137 | 76.1 | 39 | 65.0 |  | 1.00 |  |  |  | 1.00 |  |  |  |
| Yes | 43 | 23.9 | 21 | 35.0 | 0.0954 | 1.72 | 0.0939 | 0.90 | 3.21 | 1.89 | 0.0639 | 0.96 | 3.70 |
| **Q5G** |  |  |  |  |  |  |  |  |  |  |  |  |  |
| No | 102 | 56.7 | 35 | 58.3 |  | 1.00 |  |  |  | 1.00 |  |  |  |
| Yes | 78 | 43.3 | 25 | 41.7 | 0.8808 | 0.93 | 0.8213 | 0.51 | 1.68 | 0.99 | 0.9679 | 0.53 | 1.83 |
| **Q5H** |  |  |  |  |  |  |  |  |  |  |  |  |  |
| No | 48 | 26.7 | 12 | 20.0 |  | 1.00 |  |  |  | 1.00 |  |  |  |
| Yes | 132 | 73.3 | 48 | 80.0 | 0.3895 | 1.45 | 0.3035 | 0.73 | 3.08 | 1.43 | 0.3435 | 0.70 | 3.10 |
| **Q5SUM*** | 4 |  | 4 |  | 0.4190 | 1.06 | 0.4174 | 0.92 | 1.21 | 1.08 | 0.3262 | 0.93 | 1.25 |
| (Interquartile Range) | (3-6) |  | (3-6.25) |  |  |  |  |  |  |  |  |  |  |
| **Q6** |  |  |  |  |  |  |  |  |  |  |  |  |  |
| No | 158 | 87.8 | 55 | 91.7 |  | 1.00 |  |  |  | 1.00 |  |  |  |
| Yes | 22 | 12.2 | 5 | 8.3 | 0.4869 | 0.65 | 0.4119 | 0.21 | 1.68 | 0.71 | 0.5322 | 0.22 | 1.93 |
| **Q7** |  |  |  |  |  |  |  |  |  |  |  |  |  |
| No | 24 | 13.3 | 9 | 15.0 |  | 1.00 |  |  |  | 1.00 |  |  |  |
| Yes | 156 | 86.7 | 51 | 85.0 | 0.8288 | 0.87 | 0.7456 | 0.39 | 2.09 | 1.03 | 0.9440 | 0.44 | 2.59 |
| **Q8** |  |  |  |  |  |  |  |  |  |  |  |  |  |
| No | 39 | 21.7 | 14 | 23.3 |  | 1.00 |  |  |  | 1.00 |  |  |  |
| Yes | 141 | 78.3 | 46 | 76.7 | 0.8576 | 0.91 | 0.7876 | 0.46 | 1.87 | 0.84 | 0.6310 | 0.41 | 1.78 |
| **Q9** |  |  |  |  |  |  |  |  |  |  |  |  |  |
| No | 32 | 17.8 | 19 | 31.7 |  | 1.00 |  |  |  | 1.00 |  |  |  |
| Yes | 148 | 82.2 | 41 | 68.3 | 0.0288 | 0.47 | 0.0246 | 0.24 | 0.92 | 0.50 | 0.0523 | 0.25 | 1.02 |
| **Q10** |  |  |  |  |  |  |  |  |  |  |  |  |  |
| No | 15 | 8.3 | 2 | 3.3 |  | 1.00 |  |  |  | 1.00 |  |  |  |
| Yes | 165 | 91.7 | 58 | 96.7 | 0.2530 | 2.64 | 0.2069 | 0.71 | 17.05 | 1.86 | 0.4275 | 0.48 | 12.28 |
| **Q11** |  |  |  |  |  |  |  |  |  |  |  |  |  |
| No | 124 | 68.9 | 41 | 68.3 |  | 1.00 |  |  |  | 1.00 |  |  |  |
| Yes | 56 | 31.1 | 19 | 31.7 | 1.0000 | 1.03 | 0.9359 | 0.54 | 1.91 | 1.12 | 0.7279 | 0.57 | 2.16 |
| **Q12** |  |  |  |  |  |  |  |  |  |  |  |  |  |
| No | 6 | 3.3 | 2 | 3.3 |  | 1.00 |  |  |  | 1.00 |  |  |  |
| Yes | 174 | 96.7 | 58 | 96.7 | 1.0000 | 1.00 | 1.0000 | 0.22 | 6.95 | 1.13 | 0.8877 | 0.23 | 8.41 |
| **Q13** |  |  |  |  |  |  |  |  |  |  |  |  |  |
| No |  |  |  |  |  |  |  |  |  |  |  |  |  |
| Yes | 180 | 100 | 60 | 100 |  |  |  |  |  |  |  |  |  |
| **Q14** |  |  |  |  |  |  |  |  |  |  |  |  |  |
| No | 75 | 41.7 | 29 | 48.3 |  | 1.00 |  |  |  | 1.00 |  |  |  |
| Yes | 105 | 58.3 | 31 | 51.7 | 0.3717 | 0.76 | 0.3674 | 0.42 | 1.38 | 0.81 | 0.5045 | 0.44 | 1.51 |
| **Q15** |  |  |  |  |  |  |  |  |  |  |  |  |  |
| No | 11 | 6.1 | 3 | 5.0 |  | 1.00 |  |  |  | 1.00 |  |  |  |
| Yes | 169 | 93.9 | 57 | 95.0 | 1.0000 | 1.24 | 0.7509 | 0.37 | 5.61 | 0.86 | 0.8301 | 0.24 | 4.13 |
| **Q16** |  |  |  |  |  |  |  |  |  |  |  |  |  |
| No | 0 | 0.0 | 4 | 6.7 |  | 1.00 |  |  |  | 1.00 |  |  |  |
| Yes | 180 | 100.0 | 56 | 93.3 | 0.0036 | 0.01 | 0.0000 | 0.00 | 6487.54 | 0.01 | 0.0000 | 0.00 | 2979.95 |
| **Q17** |  |  |  |  |  |  |  |  |  |  |  |  |  |
| No | 90 | 50.0 | 32 | 53.3 |  | 1.00 |  |  |  | 1.00 |  |  |  |
| Yes | 90 | 50.0 | 28 | 46.7 | 0.7657 | 0.87 | 0.6548 | 0.49 | 1.57 | 0.87 | 0.6569 | 0.47 | 1.61 |
| **Q18** |  |  |  |  |  |  |  |  |  |  |  |  |  |
| No | 23 | 12.8 | 5 | 8.3 |  | 1.00 |  |  |  | 1.00 |  |  |  |
| Yes | 157 | 87.2 | 55 | 91.7 | 0.4869 | 1.61 | 0.3567 | 0.63 | 4.98 | 1.54 | 0.4175 | 0.58 | 4.89 |
| **Q19*** |  |  |  |  |  |  |  |  |  |  |  |  |  |
| 4 | 24 | 13.3 | 8 | 13.3 |  | 1.00 |  |  |  | 1.00 |  |  |  |
| 5 | 47 | 26.1 | 25 | 41.7 |  | 1.60 | 0.3277 | 0.64 | 4.26 | 1.51 | 0.4113 | 0.58 | 4.23 |
| 7 | 62 | 34.4 | 17 | 28.3 |  | 0.82 | 0.6911 | 0.32 | 2.24 | 0.94 | 0.9081 | 0.35 | 2.70 |
| 8 | 47 | 26.1 | 10 | 16.7 | 0.1311 | 0.64 | 0.4028 | 0.22 | 1.87 | 0.68 | 0.4976 | 0.23 | 2.10 |
| **Q20*** |  |  |  |  |  |  |  |  |  |  |  |  |  |
| 0 | 106 | 58.9 | 45 | 75.0 |  | 1.00 |  |  |  | 1.00 |  |  |  |
| 1 | 74 | 41.1 | 15 | 25.0 | 0.0305 | 0.48 | 0.0271 | 0.24 | 0.90 | 0.52 | 0.0606 | 0.26 | 1.01 |
| **Q21*** |  |  |  |  |  |  |  |  |  |  |  |  |  |
| 0 | 99 | 55.0 | 41 | 68.3 |  | 1.00 |  |  |  | 1.00 |  |  |  |
| 1 | 54 | 30.0 | 16 | 26.7 |  | 0.72 | 0.3245 | 0.36 | 1.37 | 0.69 | 0.2974 | 0.33 | 1.37 |
| 2 | 27 | 15.0 | 3 | 5.0 | 0.0682 | 0.27 | 0.0387 | 0.06 | 0.81 | 0.26 | 0.0393 | 0.06 | 0.82 |
| **Q22** |  |  |  |  |  |  |  |  |  |  |  |  |  |
| No | 69 | 38.3 | 19 | 31.7 |  | 1.00 |  |  |  | 1.00 |  |  |  |
| Yes | 111 | 61.7 | 41 | 68.3 | 0.4395 | 1.34 | 0.3543 | 0.73 | 2.54 | 1.14 | 0.6916 | 0.60 | 2.22 |
| **Q23** |  |  |  |  |  |  |  |  |  |  |  |  |  |
| Visit a GP | 95 | 52.8 | 23 | 38.3 |  | 1.00 |  |  |  | 1.00 |  |  |  |
| Self-medicate | 61 | 33.9 | 28 | 46.7 |  | 1.90 | 0.0496 | 1.00 | 3.62 | 2.07 | 0.0346 | 1.06 | 4.12 |
| Visit Hospital | 6 | 3.3 | 1 | 1.7 |  | 0.69 | 0.7354 | 0.04 | 4.31 | 0.45 | 0.4796 | 0.02 | 3.03 |
| Visit Polyclinic | 18 | 10.0 | 8 | 13.3 | 0.1836 | 1.84 | 0.2098 | 0.68 | 4.65 | 1.81 | 0.2570 | 0.63 | 4.94 |
| **Q24** |  |  |  |  |  |  |  |  |  |  |  |  |  |
| No | 6 | 3.3 | 2 | 3.3 |  | 1.00 |  |  |  | 1.00 |  |  |  |
| Yes | 174 | 96.7 | 58 | 96.7 | 1.0000 | 1.00 | 1.0000 | 0.22 | 6.95 | 0.83 | 0.8231 | 0.17 | 5.95 |
| **Q25** |  |  |  |  |  |  |  |  |  |  |  |  |  |
| No | 29 | 16.1 | 16 | 26.7 |  | 1.00 |  |  |  | 1.00 |  |  |  |
| Yes | 151 | 83.9 | 44 | 73.3 | 0.0855 | 0.53 | 0.0725 | 0.27 | 1.08 | 0.60 | 0.1753 | 0.29 | 1.28 |
| **Q26** |  |  |  |  |  |  |  |  |  |  |  |  |  |
| No | 7 | 3.9 | 2 | 3.3 |  | 1.00 |  |  |  | 1.00 |  |  |  |
| Yes | 173 | 96.1 | 58 | 96.7 | 1.0000 | 1.17 | 0.8446 | 0.27 | 8.02 | 1.02 | 0.9768 | 0.23 | 7.20 |
| **Q27** |  |  |  |  |  |  |  |  |  |  |  |  |  |
| No | 80 | 44.4 | 22 | 36.7 |  | 1.00 |  |  |  | 1.00 |  |  |  |
| Yes | 100 | 55.6 | 38 | 63.3 | 0.3657 | 1.38 | 0.2922 | 0.76 | 2.55 | 1.48 | 0.2204 | 0.80 | 2.82 |
| **Q28** |  |  |  |  |  |  |  |  |  |  |  |  |  |
| No | 18 | 10.0 | 8 | 13.3 |  | 1.00 |  |  |  | 1.00 |  |  |  |
| Yes | 162 | 90.0 | 52 | 86.7 | 0.4768 | 0.72 | 0.4733 | 0.31 | 1.85 | 0.83 | 0.6950 | 0.33 | 2.24 |
| **Q29** |  |  |  |  |  |  |  |  |  |  |  |  |  |
| No | 21 | 11.7 | 12 | 20.0 |  | 1.00 |  |  |  | 1.00 |  |  |  |
| Yes | 159 | 88.3 | 48 | 80.0 | 0.1290 | 0.53 | 0.1085 | 0.25 | 1.18 | 0.55 | 0.1513 | 0.24 | 1.28 |
| **Q30** |  |  |  |  |  |  |  |  |  |  |  |  |  |
| No | 47 | 26.1 | 10 | 16.7 |  | 1.00 |  |  |  | 1.00 |  |  |  |
| Yes | 133 | 73.9 | 50 | 83.3 | 0.1625 | 1.77 | 0.1400 | 0.86 | 3.95 | 1.76 | 0.1630 | 0.82 | 4.05 |
| **Q31** |  |  |  |  |  |  |  |  |  |  |  |  |  |
| No | 95 | 52.8 | 34 | 56.7 |  | 1.00 |  |  |  | 1.00 |  |  |  |
| Yes | 85 | 47.2 | 26 | 43.3 | 0.6551 | 0.85 | 0.6010 | 0.47 | 1.54 | 0.80 | 0.4774 | 0.43 | 1.48 |
| **Q32** |  |  |  |  |  |  |  |  |  |  |  |  |  |
| No | 3 | 1.7 | 1 | 1.7 |  | 1.00 |  |  |  | 1.00 |  |  |  |
| Yes | 177 | 98.3 | 59 | 98.3 | 1.0000 | 1.00 | 1.0000 | 0.13 | 20.43 | 1.03 | 0.9821 | 0.12 | 21.99 |
| **Q33** |  |  |  |  |  |  |  |  |  |  |  |  |  |
| No | 4 | 2.2 | 2 | 3.3 |  | 1.00 |  |  |  | 1.00 |  |  |  |
| Yes | 176 | 97.8 | 58 | 96.7 | 0.6416 | 0.66 | 0.6354 | 0.13 | 4.84 | 0.63 | 0.6249 | 0.11 | 5.16 |
| **Q34** |  |  |  |  |  |  |  |  |  |  |  |  |  |
| No | 10 | 5.6 | 2 | 3.3 |  | 1.00 |  |  |  | 1.00 |  |  |  |
| Yes | 170 | 94.4 | 58 | 96.7 | 0.7352 | 1.71 | 0.4987 | 0.43 | 11.31 | 1.14 | 0.8710 | 0.27 | 7.83 |
| **Q35** |  |  |  |  |  |  |  |  |  |  |  |  |  |
| No | 24 | 13.3 | 10 | 16.7 |  | 1.00 |  |  |  | 1.00 |  |  |  |
| Yes | 156 | 86.7 | 50 | 83.3 | 0.5255 | 0.77 | 0.5222 | 0.35 | 1.79 | 0.99 | 0.9805 | 0.43 | 2.41 |
| **Q36** |  |  |  |  |  |  |  |  |  |  |  |  |  |
| No | 7 | 3.9 | 1 | 1.7 |  | 1.00 |  |  |  | 1.00 |  |  |  |
| Yes | 173 | 96.1 | 59 | 98.3 | 0.6832 | 2.39 | 0.4203 | 0.41 | 45.10 | 2.50 | 0.4012 | 0.42 | 47.97 |
| **Q37** |  |  |  |  |  |  |  |  |  |  |  |  |  |
| No | 4 | 2.2 | 2 | 3.3 |  | 1.00 |  |  |  | 1.00 |  |  |  |
| Yes | 176 | 97.8 | 58 | 96.7 | 0.6416 | 0.66 | 0.6354 | 0.13 | 4.84 | 0.83 | 0.8431 | 0.14 | 6.62 |
| **Q38** |  |  |  |  |  |  |  |  |  |  |  |  |  |
| No | 3 | 1.7 | 2 | 3.3 |  | 1.00 |  |  |  | 1.00 |  |  |  |
| Yes | 177 | 98.3 | 58 | 96.7 | 0.6012 | 0.49 | 0.4428 | 0.08 | 3.80 | 0.60 | 0.6075 | 0.09 | 5.16 |
| **Q39** |  |  |  |  |  |  |  |  |  |  |  |  |  |
| No | 42 | 23.3 | 16 | 26.7 |  | 1.00 |  |  |  | 1.00 |  |  |  |
| Yes | 138 | 76.7 | 44 | 73.3 | 0.6047 | 0.84 | 0.6017 | 0.43 | 1.67 | 0.66 | 0.2559 | 0.33 | 1.36 |
| **Q40** |  |  |  |  |  |  |  |  |  |  |  |  |  |
| No | 13 | 7.2 | 3 | 5.0 |  | 1.00 |  |  |  | 1.00 |  |  |  |
| Yes | 167 | 92.8 | 57 | 95.0 | 0.7669 | 1.48 | 0.5523 | 0.46 | 6.62 | 1.29 | 0.7137 | 0.37 | 6.02 |
| **Q41** |  |  |  |  |  |  |  |  |  |  |  |  |  |
| No | 6 | 3.3 | 1 | 1.7 |  | 1.00 |  |  |  | 1.00 |  |  |  |
| Yes | 174 | 96.7 | 59 | 98.3 | 0.6837 | 2.03 | 0.5149 | 0.34 | 38.84 | 1.33 | 0.7970 | 0.21 | 26.15 |
| **Q42** |  |  |  |  |  |  |  |  |  |  |  |  |  |
| No | 43 | 23.9 | 16 | 26.7 |  | 1.00 |  |  |  | 1.00 |  |  |  |
| Yes | 137 | 76.1 | 44 | 73.3 | 0.7296 | 0.86 | 0.6654 | 0.45 | 1.72 | 0.92 | 0.8063 | 0.46 | 1.88 |
| **Q43** |  |  |  |  |  |  |  |  |  |  |  |  |  |
| No | 1 | 0.6 | 2 | 3.3 |  | 1.00 |  |  |  | 1.00 |  |  |  |
| Yes | 179 | 99.4 | 58 | 96.7 | 0.1551 | 0.16 | 0.1402 | 0.01 | 1.72 | 0.11 | 0.0741 | 0.00 | 1.18 |
| **Q44** |  |  |  |  |  |  |  |  |  |  |  |  |  |
| No | 4 | 2.2 | 2 | 3.3 |  | 1.00 |  |  |  | 1.00 |  |  |  |
| Yes | 176 | 97.8 | 58 | 96.7 | 0.6416 | 0.66 | 0.6354 | 0.13 | 4.84 | 0.78 | 0.7935 | 0.13 | 6.31 |
| **Q45** |  |  |  |  |  |  |  |  |  |  |  |  |  |
| No | 19 | 10.6 | 5 | 8.3 |  | 1.00 |  |  |  | 1.00 |  |  |  |
| Yes | 161 | 89.4 | 55 | 91.7 | 0.8048 | 1.30 | 0.6201 | 0.49 | 4.06 | 1.36 | 0.5762 | 0.49 | 4.43 |

CI = Confidence interval, OR = Odds ratio

*Each variable in these questions were given a score. Question 4: No=0, Water=1, Soap=2, Q4SUM is the summation of these scores to give each individual a combined score for the entire question. Question 5: No=0, Yes=1, Q5SUM is the summation of these scores to give an entire question score. Question 19: good recommendation=0, NEA grading=1, the cleanliness of the stall=2. Question 20 follows the same scoring as the previous question. Question 21: a score of 1 was given for each method of giving feedback.

+Age, marital status and having flu over the last 6 months were used to calculate the adjusted OR in the multivariate logistic regression model.
